# Factors associated with psychological distress during genetic counseling in high-risk women with breast cancer in Turkey

**DOI:** 10.1007/s00520-024-08573-5

**Published:** 2024-05-16

**Authors:** Dilek Anuk, Seref Bugra Tuncer, Mine Özkan, Hülya Yazıcı

**Affiliations:** 1 Division of Psychosocial Oncology and Education, Department of Preventive Oncology, Oncology Institute, Istanbul University, 34093 Istanbul, Turkey; 2 Division of Cancer Genetics, Department of Basic Oncology, Institute of Oncology, Istanbul University, 34093 Istanbul, Turkey; 3 Division of Consultation Liaison Psychiatry, Istanbul Faculty of Medicine, Istanbul University, 34093 Istanbul, Turkey; 4 Department of Medical Biology, İstanbul Arel University, 34010 Istanbul, Turkey

**Keywords:** Breast cancer, Genetic counselling, Perception of cancer, Anxiety, Depression, Health motivation

## Abstract

**Purpose:**

This study aims to shed light on the rather neglected area of research of psychological distress in women facing genetic counselling in Turkey, where few institutions providing such counselling exist.

**Methods:**

105 breast cancer patients presenting for genetic testing completed a sociodemographic and clinical questionnaire as well as validated structured questionnaires including the Beck Depression Inventory (BDI), the State–Trait Anxiety Inventory (STAI-S/T) and the Health Motivation Sub-dimension of Champion’s Health Belief Model Scale.

**Results:**

69.5% of the participants had lost a family member from cancer; 80% said the term “cancer” elicited negative thoughts (e.g., death, fear, and incurable disease). 62.9% and 37.1% attributed cancer to stress or sorrow, and genetic susceptibility, respectively. There was a negative association between health motivation and BDI scores (r:-0.433, p < 0.001). Married individuals had higher BDI and STAI-S scores (p = 0.001, p = 0.01 respectively), as well as lower STAI-T scores (p = 0.006). BDI, STAI-S and STAI-T scores were higher in those refusing genetic testing (p < 0.001, p < 0.001, p = 0.003 respectively) and those with metastases (p = 0.03, p = 0.01, p = 0.03 respectively). Furthermore, individuals with low health motivation were more likely to exhibit high BDI scores (p < 0.001) and low STAI-T scores (p = 0.02).

**Conclusion:**

Common perceptions and beliefs about cancer and genetic testing during genetic counselling were found to have a negative impact on distress in high-risk women with breast cancer. The negative relationship between psychological distress and health motivation may reduce patients' compliance with genetic counselling recommendations. A comprehensive psychological evaluation should be considered as an important part of genetic counselling.

## Introduction

Detected most frequently in women and often resulting in death, breast cancer is a significant health issue around the world [1, 2]. Each year, approximately 1 million women are diagnosed with breast cancer worldwide, with its incidence accounting for 18% of all cases of cancer [3]. The report Global Cancer Statistics 2020 puts the incidence of breast cancer in Turkey at 10.6%. It states that approximately one in four women (24.4%) in Turkey will be diagnosed with breast cancer, with 4.7% of them dying from the disease, which is the second most common cause of death [4].

Although the etiology of breast cancers varies, hereditary breast cancers constitute 10–15% of all breast cancers and 25% of primary breast cancers diagnosed before the age of 30 [1]. In the Turkish population, the prevalence of *BRCA1/2* mutations in high-risk breast carcinoma patients has been reported to vary between 19 and 37% [5]. In individuals without a cancer diagnosis but with a known BRCA1/2 pathogenic variant in their family, the overall mutation rate is 23.9% [6]. *BRCA1* and *BRCA2* gene analyses are the most commonly performed tests used to determine the inheritance of breast cancer and are usually recommended for patients at high risk of cancer and their relatives, especially if there is a family history of cancer.

Genetic testing has important implications for women's personal, family and social lives [7]. Individuals presenting for genetic testing are confronted with information about the possible genetic nature of their disease; they face the risk and the uncertainty of developing a secondary cancer; they have to make decisions about preventive surgical interventions offered to them as part of risk reduction strategies; and they have to consider the moral "obligation" to share the genetic test result with their relatives. The possibility that genetic testing processes can have psychological outcomes has often led to studies assessing anxiety, depression and cancer-related concerns in clients [8]. It has already been reported that approximately 20–55% of women with breast cancer experience psychological distress at some point in their disease, regardless of its stage and the kind of treatment being received [9–13]. Many authors have shown that the effect of genetic testing on anxiety and distress is weak and that there is no significant difference in psychological impact between positive and negative test results [14–16]. Oliveira et al. (2021) reported in their study of 178 patients having either breast or ovarian cancer who applied for genetic testing that the severity of their anxiety was greater than that of their depression, and that the presence of *BRCA1/2* mutation did not affect either anxiety or depression levels [17]. Brédart et al. (2022) psychologically evaluated breast cancer patients who had applied to genetic counselling before and after genetic testing and reported that the current distress levels of the patients did not change in the process, with 20–42% of them needing psychological support [18]. These results show that genetic testing for *BRCA* does not produce negative psychological responses. On the other hand, it is suggested that depression and anxiety experienced during the genetic testing process may be attributed to cancer-specific emotional difficulties [19].

Genetic counselling in women with cancer means that women have to face some emotional difficulties associated with the risk of developing secondary cancers, and of passing on the propensity to develop cancer to their children, as well as future generations. During cancer genetics counselling, the emphasis is usually on providing biomedical information, leaving less time to devote to addressing clients' concerns about genetic testing [20]. Although emotional factors are strong predictors of psychological distress after genetic testing [21], referral of patients for psychological counselling is more often done taking into account the impact of the genetic test result [22]. However, it has been shown that genetic testing has psychological repercussions not only associated with positive test results. Indeed, a previous diagnosis of cancer, a family history of cancer and having children predicted an even higher psychological impact [23]. Factors having major ramifications for psychological distress include being at a young age, having a diagnosis of less than one year, having inadequate social support, being single or who are highly distressed prior to approach for genetic counselling [19].

Studies comparing individuals affected and unaffected by oncological disease and those with positive, negative or uncertain outcomes [24] have shown that distress levels influence genetic testing decision-making, risk reduction decisions and screening adherence [25]. On the other hand, it has been reported that the provision of appropriate psychological support and counselling during genetic counselling, focusing on beliefs about the disease and its controllability, promotes sound medical decision-making [26]. Shiloh (2006) has been conceptualized genetic counselling in terms of self-regulation theory and developed a self-regulation model of genetic counselling by reviewing research on patients' cognitions of the genetic causality of disease and specific genetic conditions [27]. According to this model, genetic counselling is influenced by individuals' illness cognitions, risk perceptions, negative affect, as well as counselling-related decisions, which are interrelated and influenced by background factors. Shiloh (2006) suggests that disease-related negative reactions, such as fear, anxiety and depression, interact with disease cognitions, risk perception, family's approach to the test/test result, personality traits, perceived impact of genetic testing and decision about genetic counselling [27]. Illness cognitions include identity (impression of the disease, experience with the disease, signs and symptoms, and family history of the disease), timeline (uncertainty about the occurrence and duration of the disease), outcomes (quality of life and life expectancy), cause of the disease (genetic, stress, chance, etc.) and control cognitions (will preventive medicine or preventive health behaviors be useful in controlling the condition or is it all down to luck?) [27]. In this context, there are almost no data on how women with genetically high-risk breast cancer feel about genetic testing, or on their level of psychological distress and related variables in Turkey. Therefore, we aimed to investigate (i) perceptions of genetic testing, (ii) depression and anxiety levels, (iii) sociodemographic and cancer-related variables such as cancer stage, metastasis or recurrence, family history of cancer and death from cancer that may be associated with depression and anxiety scores, and (iv) the relationship between depression and anxiety levels and health motivation in genetically high-risk Turkish breast cancer patients presenting for genetic testing. This study has three hypotheses:

- Hypothesis 1 (H1): There is a positive relationship between levels of depression and anxiety in high-risk Turkish breast cancer patients presenting for genetic testing and socio-demographic variables such as young age, high education level, lower income and having children.
- Hypothesis 2 (H2): There is a positive relationship between levels of depression and anxiety in high-risk Turkish breast cancer patients presenting for genetic testing and such negative cancer experiences as advanced stage cancer, cancer recurrence, family history of cancer, and familial history of death from cancer.
- Hypothesis 3 (H3): There is a negative relationship between depression and anxiety and health motivation in high-risk Turkish women with breast cancer presenting for genetic testing.

## Methods

### Study design and participants

The sample consisted of 105 breast cancer patients referred to genetic testing at the cancer genetics outpatient clinic of a leading oncology center in Turkey between August 2017 and March 2019. Genetic counselling was done by a multidisciplinary team consisting of biologists specializing in cancer genetics, as well as a psychologist specializing in psycho-oncology and a psychiatrist. Data for the study were gathered during the patients' first genetic counselling session when they applied for genetic testing. The objectives of the first genetic counselling interview were as follows [28].

1. To interpret family and medical histories in order to evaluate the probability of disease occurrence or recurrence.
2. To provide education related to inheritance, testing, management, prevention, resources and research.
3. To offer counselling promoting informed decision-making and adjustment to the risk or circumstance.

During the first genetic counselling interview at our institution, information is collected about the patient’s socio-demographic characteristics, along with the medical history of the patient and his or her family. If the patient agrees, a family tree is drawn up. Patients are informed about available genetic testing and its suitability for their needs. Blood samples are not taken immediately so as to give the patient time to decide about genetic testing. Once the patient chooses to undergo genetic testing, they are provided with contact information to make an appointment. At the genetic counselling outpatient clinic, the psycho-oncologist participates in the initial consultation as a member of the team. The patient may also be invited for a comprehensive psychological assessment if considered necessary. If the patient requires psychological treatment, the psycho-oncologist will refer them to the psycho-oncology outpatient clinic, which is conducted jointly with a psychiatrist.

Participants identified as genetically high risk either had a first-degree relative with premenopausal breast/ovarian cancer (27.8%) or were diagnosed with breast cancer before the age of 50 (19.7%), or both conditions were present simultaneously (52.5%). The exclusion criteria were: being younger than 18 years of age, not being literate, not having been diagnosed with breast and/or ovarian cancer. In addition, chronic mental illnesses such as schizophrenia, psychotic disorder or cognitive impairment were determined as exclusion criteria, as it is thought that they may adversely affect the mental or cognitive capacity of the individuals and cause them to fill in the scales in a healthy way. On the other hand, the presence of diagnoses such as non-psychotic anxiety or depressive disorders that existed before the diagnosis of cancer was included as a variable in terms of the psychological distress of the participants in the genetic counselling process. Relevant data were obtained from the participants before the first genetic counselling interview. After the interview, they were asked if they were satisfied with the genetic counselling and what their decisions were regarding genetic testing. Patients thought to be in need of psychological treatment were referred to the psycho-oncology outpatient clinic for psychological evaluation. This study complied with the tenets of the Declaration of Helsinki and Istanbul University Oncology Institute’s Academic Board (no. 461833) approved the study’s ethical standards. Patients were informed about the study and 105 of 129 patients gave their written consent.

### Measures

#### Semi-structured Interview Form

This data collection tool was developed by the researchers based on the findings of previous studies [22, 26]. It consists of three parts: sociodemographic and cancer-related data, genetic test-related data, and thoughts about cancer. Sociodemographic and cancer-related data included questions about when the disease was diagnosed, the stage of cancer, the treatments received and the presence of recurrence and/or metastasis, the presence of a family history of cancer, cancer-related deaths in the family, and whether there was a history of psychiatric illness. The data related to genetic testing included questions about the factor of genetic predisposition, the person referring the patient for testing, the reason for testing given to the patient when the patient was sent for testing, and the patient's own reason for getting the testing done. In order to understand what the patient thought about cancer, there were questions about the meaning of cancer, the cause of cancer, whether cancer had been treated, and whether cancer could be treated in the future.

#### Beck Depression Inventory

The Beck Depression Inventory (BDI), developed by Beck et al. (1961), is used to evaluate the level of depression [29], with higher total scores indicating greater severity. In our study, we used the Turkish-translated BDI, the reliability and validity of which were evaluated by Teğin (1980); scores of 17 or greater on it were defined as signifying severe depression [30]. Cronbach's alpha values of the BDI were found to be 0.80. In the current study, the Cronbach's alpha for the BDI was 0.82.

#### State–Trait Anxiety Inventory

The State-Trait Anxiety Inventory (STAI) is a 40-item self-reported questionnaire designed by Spielberg et al. [31] to measure both state and trait anxiety. Trait anxiety (STAI-T) refers to relatively stable individual differences in anxiety proneness, i.e., to differences between people in the tendency to perceive stressful situations as dangerous or threatening and to respond to such situations with elevations in the intensity of their state anxiety (STAI-S) reactions. In the Turkish adaption study, anxiety is measured on a scale of 20 to 80. A score of 40 or higher indicates high anxiety. Additionally, the STAI-S and STAI-T both exhibit high Cronbach's alpha values of 0.83 and 0.87, respectively [32]. In the current study, the Cronbach's alpha for the STAI-S and STAI-T subscales were 0.82 and 0.77, respectively.

#### Health Motivation Sub-dimension of Champion’s Health Belief Model Scale

Champion’s Health Belief Model Scale was developed in 1993 by Victoria Champion [33]. The health motivation (HM) subdimension of the scale aims to evaluate the beliefs and behaviors of the individual essential for being healthy. The scale was generally used in the studies on preventive health such as breast self-evaluation, and mammography. The HM scale, which may have a determining effect on compliance with additional treatment recommendations as a result of genetic testing, was used to evaluate the relationship between health motivation and psychological distress in this study. The Cronbach's alpha value of the health motivation subdimension of the scale was reported to be 0.83 in the study of the adaptation of the scale into Turkish [34]. In the current study, the Cronbach's alpha for the health motivation subscale was 0.82.

### Statistical Analysis

The statistical analysis of the data used in this study was performed using the Statistical Package for the Social Sciences version 22.0. Firstly, the necessary assumptions were tested in order to decide which tests (parametric/non-parametric) to use. Kolmogorov–Smirnov, Shapiro–Wilk tests, kurtosis and skewness values and histogram plots were used to determine the normality of the distribution. Since the kurtosis and skewness values were within ± 2.0, the values were considered to be normally distributed. When comparing two independent groups, the independent sample t-test was used for normally distributed data and the Mann–Whitney U test for non-normally distributed data. The continuous variable age was transformed into a categorical variable as ≤ 42 and 43 < by taking the mean age (43.1) as reference. One-way analysis of variance was used for normally distributed data when comparing more than two unrelated groups. The Bonferroni test, one of the post hoc tests, was used to determine the source of the difference. Levene's statistic was used to determine homogeneity of variances and it was found that the variances were homogeneous. For data that did not show a normal distribution, the Kruskal–Wallis H test and pairwise comparisons were used to determine the source of the difference. The relationship between variables was analysed using Pearson's correlation coefficient.

Multiple linear regressions were performed in order to determine the variables associated with depression and anxiety in high-risk breast cancer patients who applied for genetic counselling. The regression models included BDI, STAI-S, and STAI-T as dependent variables. Among the independent variables, only health motivation was a continuous variable. To use categorical variables in regression analysis, dummy variables were created with values of 0 and 1 and included in the analysis. Initially, the regression models included all variables. The backward elimination method was then used to identify the estimators that best fit the models. Prior to analysis, the assumptions required for multiple linear regression analysis were checked. To test for multivariate outliers, Mahalanobis distance, Cook's distance, and Centered Leverage distance were used. Two cases exceeded two standard deviations and were labeled as potential multivariate outliers in BDI. However, they were not removed as the other two distances did not show any violation. The Durbin-Watson value was calculated to determine the presence of autocorrelation, and it was found to be 1.969, 1.872, and 1.678, which is close to the desirable value of 2. Therefore, there is no autocorrelation problem with the values. The VIF values of the independent variables ranged from 1.077 to 2.883, indicating the absence of multicollinearity in the dataset since no VIF value exceeded 5. Furthermore, the correlation coefficients between the predictor variables and BDI ranged from 0.216 to -0.489, and with STAI-S from 0.083 to 0.542, and and with STAI-T from 0.017 to 0.431. These correlation coefficients suggest that there is no multicollinearity problem as they are all less than 0.80. The results were assessed at a 95% confidence interval with significance set at *p* < 0.05.

## Results

### Sociodemographic and cancer-related characteristics of participants

The sociodemographic and cancer-related characteristics of participants are shown in Table 1. Their mean age was 43.1 ± 10.6 years (Range: 19–71 years). Most participants reported being married (74.3%), having a moderate income (69.5%), and having had received a diagnosis of breast cancer, 4.8% of whom had been diagnosed with both breast and ovarian cancer. Only 2.9% of the patients had not started to receive any treatment, and the remaining (97.1%) had received a treatment protocol consisting of surgery, chemotherapy, and radiotherapy, alone or in combination. More than a half (54.3%) of participants had either stage 1 or stage 2 cancer, 19% had had a recurrence, 21.9% had experienced a metastasis, the majority (82.9%) had a family history of cancer, 69.5% had lost first- or second-degree relatives from cancer, and 20% had received psychiatric treatment.

**Table 1:** Sociodemographic and cancer-related characteristics of patients

	n (105)	**%**
Age (years)		
Mean ± SD (Range)	43,1 ± 10,64 (19–71)	
Duration of cancer (month):		
Mean ± SD (Range)	20.4 ± 18.1 (1–64)	
Education level		
Literate / elementary school	28	26.7
Secondary school graduates	34	32.4
College	43	41
Marital status		
Married	78	74.3
Single	18	17.1
Divorced/Widowed	9	8.6
Employment		
Working fulltime	47	44.8
Housewife	42	40
Retired	15	14.3
Student	1	1
Income level		
Low	13	12.4
Middle	73	69.5
Upper	19	18.1
Cancer site		
Breast cancer	100	95.2
Breast cancer + Over cancer	5	4.8
Stage		
Stage Ia / Ib	25	23.8
Stage II	32	30.5
Stage III	39	37.1
Stage IV	9	8.6
Oncological treatment		
Surgery + chemotherapy + radiotherapy	47	44.8
Surgery + chemotherapy	30	28.6
Surgery + radiotherapy	4	3.8
Chemotherapy + radiotherapy	5	4.8
Surgery	8	7.6
Chemotherapy	8	7.6
Treatment is not yet started	3	2.9
Recurrence		
No	85	81
Yes	20	19
Metastasis		
No	82	78.1
Yes	23	21.9
Family history of cancer		
No	18	17.1
Yes	87	82.9
Death due to cancer in the family		
No	32	30.5
Yes	73	69.5
Psychiatric history		
No	84	80
Yes	21	20

### Participants’ information and thoughts on genetic testing and cancer

Table 2 contains the information provided to the participants when referred to genetic testing and the participants’ motivations for undergoing such testing. Their oncologist or surgeon had referred most participants (91.5%) to the cancer genetics outpatient clinic to undergo genetic testing. The main reasons given by the patients for undergoing the genetic test were to take precautions for themselves or their children/relatives (53.3%) and to find out whether they had inherited their disease(s) (34.3%). When asked “what does cancer mean to you?,” 80% of the responses were as follows: death, fear, evil, untreatable disease, pain, unhappiness or hopelessness, and difficult treatment. When the participants were asked “What do you think is the cause of cancer?,” the top answers were stress or sorrow (62.9%), genetic susceptibility (37.1%), unhealthy nutrition (26.7%), smoking or alcohol consumption (6.7%), and environmental conditions such as air pollution and radiation (5.7%). While only 27.6% of participants gave positive responses to the question “Can cancer be treated at present?,” 73.3% of participants gave positive responses to the question “Do you think that cancer can be treated in the future?”. Almost all of the participants (92.4%) were satisfied with the first interview for genetic counselling; however, 5.7% of the patients did not accept the genetic test, and 10.5% stated that they were undecided. After the genetic counselling interview, which also included a psycho-oncology specialist, 24.8% of the participants were referred to the outpatient psycho-oncology clinic for psychological treatment.

**Table 2:** Information and thoughts on genetic testing and cancer

	n (105)	%
Genetically predisposition factor		
(ı) Having a first-degree relative with premenopausal breast/ovarian cancer	28	26.7
(ıı) Diagnosed with breast cancer before the age of 50	22	20.9
(ııı) Both (ı) and (ıı) were present	55	52.4
Who leads to genetic testing		
Oncologist	74	70.5
Surgeon	22	21
Her own wish	2	1.9
Others	7	6.7
Information provided while referring to the test		
Genetic examination	69	65.7
Identify risks and take precautions for the patient and family	12	11.4
To determine the appropriate treatment	10	9.5
To learn the risk of spreading the disease	6	5.7
To determine the cause of the disease	5	4.8
Other	3	2.9
The aim of the patient to have a genetic test		
To find out whether cancer is genetic	36	34.3
To learn future risks for the patient himself/for precautionary purposes	23	21.9
To learn about cancer risk or take precautions for their children	17	16.2
As a precautionary measure for other family members	16	15.2
Doctor's request	6	5.7
To determine the appropriate treatment	5	4.8
Curiosity	2	1.9
“What does cancer mean for you?”		
Negative (Death /fear/ evil or untreatable disease/ suffering pain etc.)	84	80
Positive (A second chance at life/fight and win)	12	11.4
Ordinary (Like flu, disease of our Age)	9	8.6
“What do you think is the cause of cancer?” *		
Stress/sorrow	66	62.8
Genetic susceptibility	39	37.1
Unhealthy nutrition	28	26.7
Don't know / have no idea	11	10.5
Environmental conditions (such as air pollution/ radiation)	7	6.6
Smoking/alcohol consumption	7	6.6
Fate/Destiny	1	1
“In your opinion, can cancer be cured today?”		
No	29	27.6
Yes	28	26.7
Partially	48	45.7
“Can you think that cancer will be cured in the future?”		
No	11	10.5
Yes	77	73.3
Partially	17	16.2
Satisfaction with genetic counseling:		
No	3	2.9
Yes	94	89.5
Partially	8	7.6
Accept to have the genetic test:		
No	6	5.7
Yes	88	83.8
Can’t decide	11	10.5
Referral to Psycho-oncology department		
No	79	75.2
Yes	26	24.8

^*^In this question, participants can select more than one option

### Depression, anxiety and health motivation

The mean scores and standard deviations of the participants were 12.1 ± 7.6 (0–35) on the BDI, 45.2 ± 8.9 (27–66) on the STAI-S, 41.8 ± 9.8 (20–62) on the STAI-T and 28.6 ± 5.2 (9–35) on the HM. The scores showed that 29.5% of participants had severe depression, 66.7% had high-state anxiety, and 52.4% had symptoms of high-trait anxiety. A negative correlation was found between the scores of HM and BDI, (r: -0.433. p < 0.001.). A positive correlation was identified between the scores of BDI and STAI-S, STAI-T scores (r: 0.668. p < 0.001; r: 0.695. p < 0.001 respectively).

When the relationship between depression and anxiety scores and variables was analysed, younger age, being married, low income level, presence of metastases, presence of recurrence, family history of death from cancer, desire for genetic testing for prevention (for self, children or other family members), belief that cancer is currently incurable and belief that cancer will be incurable in the future were some of the variables found to be associated with higher mean scores on the BDI, STAI-S and STAI-T. The relationships between scale scores and variables are detailed in Table 3.

**Table 3:** Variables related to participants' depression, state and trait anxiety levels

	BDI	STAI-S	STAI-T
Age:			
≤ 42	12.2 ± 7.9 t:2.634	44.7 ± 9.1 t:2.907	46.7 ± 8.5 t:1.696
43 ≤	8.3 ± 6.8 **p = 0.01**	39.3 ± 9.7 **p = 0.004**	43.8 ± 9 p > 0.05
Education			
(1) Primary school	11.7 ± 9.1 F:0.890	42.5 ± 10.8 F:0.169	48.5 ± 10.2 F: 3.741
(2) High school	9.3 ± 5.8 p > 0.05	41.1 ± 8.38 p > 0.05	45.4 ± 7.9 **p = 0.027**
(3)University	9.8 ± 7.7	41.9 ± 10.2	42.8 ± 8
Marital Status			
Not married	7.6 ± 5.7 t:2.12	37.7 ± 9.7 t:2.601	42 ± 7.4 t:2.177
Married	11.1 ± 7.9 **p = 0.04**	43.2 ± 9.4 **p = 0.01**	46.3 ± 9.1 **p = 0.03**
Income level			
(1) Low	77.7 Χ^2^_KW_:12.742	49.2 ± 1 F:4.570	49.7 ± 10.3 F:5.690
(2) Middle	52.3 **p = 0.02**	40.8 ± 9.6 **p = 0.01**	45.7 ± 8.3 **p = 0.005**
(3) Upper	38.9 **1 > 2, 3**	40.7 ± 8.1 **1 > 2, 3**	39.9 ± 7.9 **1 > 3**
Social support			
No	12.3 ± 8 t:3.998	43.5 ± 10.1 t:2.333	46.9 ± 9.1 t:2.651
Yes	6.6 ± 4.8 **p < 0.001**	39 ± 8.7 **p = 0.02**	42.3 ± 7.7 **p = 0.009**
History of psychiatric disorder			
No	9 ± 6.4 t:-3.698	41 ± 9.6 t:-1.78	43.7 ± 8.2 t:-3.885
Yes	15.8 ± 10 **p < 0.001**	45.5 ± 10 p > 0.05	52.1 ± 9 **p < 0.001**
Duration of cancer (year)			
(1) 0–1	55.5 Χ^2^_KW_:0.992	41.6 ± 9.7 F:0.023	45.3 ± 8.1 F:0.042
(2) 1–2	48.4 p > 0.05	41.9 ± 12.6 p > 0.05	45.1 ± 11.5 p > 0.05
(3) 2 ≤	50.3	42.1 ± 8.5	44.8 ± 9.1
Cancer stage			
(1) Stage 1–2	10.5 ± 7.3 F:0.321	43 ± 9.6 F:1.807	46.7 ± 8.3 F:2.672
(2) Stage 3	10.1 ± 8.3 p > 0.05	41.3 ± 9.6 p > 0.05	44.1 ± 9.5 p > 0.05
(3) Stage 4	8.3 ± 6.1	36.6 ± 10.6	40.1 ± 7.2
Metastasis			
No	45.4	39.4 ± 9.2 t:5.166	43.6 ± 8.7 t:3.466
Yes	78.7	49.9 ± 7.1 **p < 0.001**	50.4 ± 7.2 **p = 0.001**
Recurrence			
No	46.9 z:4.38	39.7 ± 9.2 t:5.247	43.9 ± 8.7 t:3.114
Yes	80.7 **p < 0.001**	51.3 ± 5.7 **p < 0.001**	50.7 ± 7.4 **p < 0.001**
Cancer history in the family			
No	7.7 ± 7 t:2.330	38.2 ± 10.6 t:2.605	42.7 ± 8.7 t:1.959
Yes	11.3 ± 7.6 **p = 0.02**	43.4 ± 9 **p = 0.01**	46.3 ± 8.8 p > 0.05
Familial history of death from cancer			
No	5.8 ± 4.6 t:4.653	36 ± 9.7 t:4.699	40.7 ± 8.2 t:3.922
Yes	12.4 ± 7.7 **p < 0.001**	44.7 ± 8.5 **p < 0.001**	47.4 ± 8.3 **p < 0.001**
The aim of having a genetic test			
Other	7.1 ± 6.2 t:4.304	37.5 ± 9.6 t:4.607	42.8 ± 8.4 t:2.654
Reduce the risks/precautions	12.9 ± 7.6 **p < 0.001**	45.6 ± 8.4 **p < 0.001**	47.3 ± 8.8 **p = 0.009**
Can cancer be cured today?			
No	13.2 ± 7.9 t:5.666	45 ± 9.1 t:4.398	47.9 ± 8.3 t:4.092
Yes	5.7 ± 3.8 **p < 0.001**	37.1 ± 8.8 **p < 0.001**	41.1 ± 8.2 **p < 0.001**
Cancer will be cured in the future?			
No	13.9 ± 6.2 t:3.112	46.8 ± 6.7 t:3.250	49.3 ± 6.1 t:2.870
Yes	8.9 ± 7.6 **p = 0.002**	40.1 ± 10.1 **p = 0.002**	43.8 ± 9.3 **p = 0.005**
Satisfaction with genetic counseling			
(1) No	29,3 ± 5,1 F:40.963	57 ± 4,6 F: 9.821	58,3 ± 9,9 F: 6.631
(2) Partially	22,9 ± 6,3 **p < 0.001**	51,5 ± 6,8 **p < 0.001**	51,6 ± 6,6 **p = 0.002**
(3)Yes	8,4 ± 5,8 **1, 2 > 3**	40,5 ± 9,2 **1, 2 > 3**	44,2 ± 8,5 **1 > 3**
Accept to have the genetic test			
(1) No	27.2 ± 6.7 F:40.866	58,8 ± 3,6 F:22.550	59,5 ± 6,6 F: 17.536
(2) Can’t decide	17.3 ± 4.9 **p < 0.001**	50,6 ± 4,9 **p < 0.001**	52,3 ± 4,9 **p < 0.001**
(3) Yes	8.1 ± 5.7 **1, 2 > 3**	39,5 ± 8, 7 **1, 2 > 3**	43,4 ± 8,1 **1, 2 > 3**
Referral to Psycho-oncology			
No	7,6 ± 5,9 t:7.540	39,6 ± 9,5 t:4.417	42,9 ± 8,3 t:5.060
Yes	18 ± 6,9 **p < 0.001**	48,6 ± 7,1 **p < 0.001**	52,1 ± 6,7 **p < 0.001**

t:Independent Sample t test

z: Man Whitney-U test

Χ^2^KW: Kruskal Wallis-H test

F: Anova

The results of the regression analysis performed to determine the possible associated factors for BDI, STAI-S and STAI-T levels are as follows (Table 4): In our sample, individuals were more likely to have higher BDI, STAI-S and lower STAI-T scores if they were married (β = 0.321, p = 0.001; β = 0.218, p = 0.01; β = 0.340, p = 0.006 respectively), refused genetic testing (β = -0.308, p < 0.001; β = -0.351, p < 0.001; β = -0.270, p = 0.003 respectively) and had metastases (β = 0.232, p = 0.03; β = 0.249, p = 0.01; β = 0.286, p = 0.03 respectively). Individuals were more likely to have higher BDI or STAI-S scores if they had at least one familial history of death from cancer (β = 0.221, p = 0.004; β = 0.255, p = 0.001 respectively) and did not believe in the possibility of a cure for cancer in the future (β = 0,150, p = 0.04; β = 0,214, p = 0.006 respectively). Being in the age group of 42 years and younger associated with the higher BDI score (β = -0.262, p = 0.001). Having only a psychiatric history increased related to the higher STAI-T score (β = 0,322, p < 0.001). A negative relationship was found between HM score and BDI score (β = -0.269, p < 0.001).On the other hand, HM scores was positively associated with STAI-T scores (β = -0,204, p = 0.02). The results showed that the models explained 60.3% of the variance of depression score (Adjusted R^2^ = 0.603, F = 23. 534, p < 0.001). 52.1% of the variance of state anxiety (R2 = 0.521, F = 19.862, p < 0.001) and 41.9% of the variance of trait anxiety (R2 = 0.351, F = 9.041, p < 0.001) in the patients included in this study. To calculate the power of the three models, a retrospective post hoc power analysis was performed using G*Power. The analysis indicates that a sample size of 105 women, a medium effect size of at least 0.15, and an α of 0.05 resulted in a power of 0.81 for 7 predictors (BDI and STAI-T) and 0.84 for 6 predictors (STAI-S).

**Table 4:** Factors related to depression, state and trait anxiety

		BDI				STAI-S					STAI-T				
	B	β	t	p	VIF	B	β	t	p	VIF	B	β	t	p	VIF
Age (0 = ≤ 42 years, 1 = ≥ 43 years) Marital Status (0 = not married, 1 = married) Psychiatric history (0 = no, 1 = yes) Metastasis (0 = no, 1 = yes) Familial history of death from cancer (0 = no, 1 = yes) The aim of having a genetic test: (0 = Other 1 = Reduce the risks/precautions) Cancer will be cured in the future? (0 = no, 1 = yes) HM (Health Motivation) Acceptance of genetic test (0 = no, 1 = yes) Social Support (0 = not enough, 1 = enough) **Constant** **R**^**2**^ **Adjusted R**^**2**^ **F** **Durbin-Watson**	-2,418 6.172 - 5.080 4.488 - -2.971 -0.424 -7.674 -	-0.262 0.321 - 0.232 0.221 - -0.150 -0.269 -0.308 -	-3.302 3.426 - 2.104 2.948 - -2.104 -4.195 -4.514 -	**0.001** **0.001** **-** **0.03** **0.004** **-** **0.04** ** < 0.001** ** < 0.001** **-**	1.649 2.295 2.883 1.466 - 1.332 1.077 1.216 -	- 4.503 - 5.919 5.626 4.861 -4.594 - -9.503 -	- 0.218 - 0.249 0.255 0.248 -0.214 - -0.351 -	- 2.487 - 2.624 3.457 3.442 -2.781 - -4.617 -	- **0.01** - **0.01** **0.001** **0.001** **0.006** **-** ** < 0.001** **-**	- 1.672 - 1.960 1.181 1.285 1.285 - 1.255 -	-8.198 9.843 7.830 - - -4.179 0.402 -8.453 -2.244	- -0.340 0.322 0.286 - - -0.168 0.204 -0.270 -0.165	- -2.839 3.814 2.179 - - -1.877 2.432 -2.994 -1.931	**0.006** ** < 0.001** **0.03** - - 0.063 **0.02** **0.003** 0.056	2.300 1.144 2.552 - - 1.291 1.122 1.307 1.165
	43.251 0.629 0.603 23.534 1.969		8.913	** < 0.001**		40.186 0.549 0.521 19.862 1.872		7.236	** < 0.001**		42.306 0.395 0.351 9.041 1.678		8.308	** < 0.001**	

β:Standardized regression coefficient,

VIF: Variance Inflation Factor

## Discussion

Most research into anxiety and depression related to hereditary breast cancer has concentrated on assessing the psychological effects of receiving genetic test results. However, the psychological impact of genetic testing may not only be tied to positive test results. Emotional difficulty triggered by a previous cancer diagnosis, having children, or the possibility of cancer in other organs may also influence the level of psychological distress experienced by individuals [21]. Given the adverse repercussions of a positive test result on patients, they are usually recommended psychological support during genetic examination [17]. However, some patients undergoing pre-testing experience psychological distress that may prevent them from receiving the support they need [35]. Research shows that individuals with increased psychological distress may have difficulty receiving and remembering information, and as a result, may find it more difficult to comply with follow-up and treatment recommendations [36]. From this perspective, a study was conducted to examine potential factors that influence anxiety and depression levels among high-risk breast cancer patients seeking genetic counselling in Turkey. To our knowledge, this is the first study on the subject in the country.

In this study, certain socio-demographic variables, including young adults’, marital status, low income, psychiatric history, and presence of metastasis/recurrence, were significantly associated with depression and anxiety scores (see Table 3). These findings are consistent with the results of prior research, indicating that young women receiving genetic testing are more likely to report psychological problems [37]. In contrast to the current body of literature, our study found a correlation between being married and higher depression and state anxiety scores. Moreover, our regression analyses indicated that married participants presented higher depression and anxiety scores, but lower trait anxiety levels (refer to Table 4). However, in line with prior research, we also observed that being single was linked to greater psychological issues in our participants, but only with regard to trait anxiety scores [19]. The association between marriage and increased levels of state anxiety and depression could be explained by the considerable burden of having to explain the likely positive results of genetic testing to their spouses, children and family. Indeed, Gomes et al. (2022) discovered that the ability of mutation carriers to adjust to this circumstance is heavily influenced by family functioning and linked to the response of family members, particularly spouses and siblings, to new genetic information [38].

A significant relationship was uncovered between cancer recurrences or metastasis and increasing depression or anxiety scores, as shown in Table 3. Regression analysis showed that the presence of metastases was likely to be associated with higher depression, state and trait anxiety scores in patients, as shown in Table 4. These findings are similar to previous studies indicating that the existence of disease recurrence or metastasis elevates the psychological symptoms of breast cancer [11, 39]. A link between psychiatric illness and depression and trait anxiety scores was found among breast cancer patients seeking genetic counselling. The regression analysis determined that the presence of a psychiatric history was only likely to increase trait anxiety (Table 4). Additionally, Okamura et al. (2005) demonstrated that breast cancer patients with a history of depression have a greater risk of suffering from psychiatric illnesses [39].

Roughly 55% of respondents were presenting for genetic testing to determine potential risks for themselves, their children or other family members, and had received relevant information about the procedure (Table 2). For instance, Claes et al. (2004) found that the primary motivation for genetic testing was related to the wellbeing of their children [40]. Hallowell et al. (2004) emphasized the importance of cancer-associated risk management in motivating individuals to undergo genetic testing [41]. In addition, in the regression analysis, it was found that patients undergoing genetic testing to take precautions against cancer may experience an increase in state anxiety scores (Table 4). Patients' experiences with cancer and the negative meanings attached to cancer by society may play an important role in patients' desire to take more precautions (41). Hence, our statistical analyses have indicated that a family history of cancer or familial history of death from cancer correlates with higher depression and anxiety scores amongst genetically high-risk breast cancer patients (Table 3). Similarly, according to the regression analysis, depression and anxiety levels are likely to increase in the presence of a family history of death from cancer. (Table 4).

When asked about the cause of cancer, the most frequently cited factors were stress and sadness, either alone or in combination with others, followed by genetic predisposition (Table 2). Despite limited clinical evidence supporting a causal connection between cancer and stress or sadness, it remains a common belief [42, 43]. Given that almost half of the participants were university graduates and were in a social environment familiar with genetic testing, it was notable that participants cited stress or sadness as a cause of cancer. Participants' attribution of cancer to an external stressor that can be changed and controlled may be due to its greater tolerance compared to attributing cancer to an internal factor, such as genetic predisposition, that cannot be controlled or changed.

Research on health motivation, a main pillar of the health belief model, has been used to assess people's motivation for self-examination, doctor's visits and screening methods such as mammography/ultrasound for cancer [44, 45]. There are studies similar to the current one that show a negative relationship between health motivation and psychological distress [46, 47]. However, these have been carried out in healthy populations. We found that the health motivation scores of patients with high scores of depression and trait anxiety were significantly lower. In addition, a significant negative correlation was observed between HM scores and BDI scores. The results of the regression analysis, revealed that health motivation was negatively associated with depression but positively associated with trait anxiety (Table 4). As depression and anxiety levels increase, patients' motivation to protect their health decreases, leading to adverse consequences for their health. Furthermore, the depression, state, and trait anxiety scores of patients who declined genetic testing or were undecided were higher, as displayed in Table 3. The results of the regression analysis suggest a relationship between not accepting genetic testing and depression, state anxiety and trait anxiety (Table 4). Taken together, healthcare providers should be aware of symptoms of depression and anxiety and take them seriously so that patients' compliance with treatment recommendations after genetic testing can be increased.

According to self-regulation model of genetic counselling,it can be said that in high-risk breast cancer patients who apply for genetic counselling, having had negative experiences with cancer (e.g., metastasis or cancer-related death in the family) and perceiving the disease as uncontrollable (i.e., as incurable in the future, resulting in a low HM level), being married (in terms of the family's anticipated response to the test result), considering to undergo genetic testing in order to take precautions for themselves, or their children or relatives (in terms of perception of risk cancer poses for them) and refusing to have genetic testing, all may have a detrimental impact on mental health (e.g., anxiety and/or depression) and the genetic counselling process as a whole.

## Conclusion and practical implications

To our knowledge, this study is the first in Turkey to psychologically assess high-risk breast cancer patients referred for genetic testing during their first genetic counselling interview. In Turkey and in countries like Turkey where genetic counselling is becoming increasingly widespread, we believe that the attributions and thoughts that constitute common illness representations in the society about cancer and genetic testing in society may be positively associated with the cancer control success and patient distress. Given the nature of genetic information and its link to health and wellness, the burden of disease, and its impact on relatives and future reproductive decisions, patients are likely to have a variety of thoughts, feelings and concerns about genetic counselling. Previous literature emphasizes that patients/consultants should be provided with sufficient opportunity to process information, reflect on options, express their values and beliefs and make informed decisions 

[47]. Therefore, it is important that the possible psychological problems of high-risk cancer patients referred for genetic testing are not evaluated solely in the context of a positive genetic test result, but that psychological assessment is included as a standard part of genetic counselling before and throughout the genetic testing process.

### Strengths and limitations

This study had a cross-sectional design. Our sample included only breast cancer patients predisposed to hereditary cancer who were referred for genetic testing. Long-term studies are needed to evaluate the effects of breast cancer patients' depression and anxiety levels in terms of accepting or complying with the preventive medical interventions recommended them. This study was carried out in one of the few centers available in Turkey. Multicenter studies can reach more participants and obtain more generalizable results.

Despite the limitations, this study identifies the variables associated with higher levels depression and anxiety in breast cancer patients predisposed to hereditary cancer. Moreover, the study evaluates the depression, anxiety and health motivation levels of the in these patients. The findings can be illuminating for interventions and programs to be developed in terms of prevention or care of cancer.

## Data Availability

The data that support the findings of this study are available from the corresponding author upon reasonable request.
